# IREB2 Knockdown Alleviates High-Fat Diet-Induced Nonalcoholic Fatty Liver Disease by TLR4/NF-κB Signaling Inactivation

**DOI:** 10.5152/tjg.2025.24054

**Published:** 2025-04-02

**Authors:** Yongmin Hu, Shengjun Zhang, Qingjian Jiang, Tengqian Chen, Jia Luo, Yigui Jiang

**Affiliations:** 1 Department of Clinical Nutrition, Sanming First Hospital Affiliated to Fujian Medical University, Sanming, China; 2 Department of Gastroenterology, Sanming First Hospital Affiliated to Fujian Medical University, Sanming, China

**Keywords:** Hepatic steatosis, nonalcoholic fatty liver, NF-κB signaling cascade, toll-like receptor 4, IREB2

## Abstract

**Background/Aims:**

Nonalcoholic fatty liver disease (NAFLD) is one of the most prevalent chronic liver diseases and is characterized by extensive deposition of fat in hepatocytes. This study aims to elucidate iron responsive element binding protein 2’s (IREB2) role in high-fat diet (HFD)-induced NAFLD and the regulatory mechanisms of the TLR4/NF-κB cascade.

**Materials and Methods:**

Male rats were fed an HFD to induce the NAFLD in vivo model. Changes in body weight and liver tissue weight were measured. Liver tissue damage and hepatic steatosis were monitored by hematoxylin–eosin staining in rats. Fat droplet size of rat liver tissue was detected by oil red O staining kit to evaluate fat deposition. Total cholesterol (TC), triglycerides (TG), alanine transaminase (ALT), Aspartate transaminase (AST), fatty acid synthase (FAS, and PPARα were detected by enzyme-linked immunosorbent assay (ELISA) kit and western blot. Interleukin-1-beta (IL-1β), interleukin-6 (IL-6), and tumor necrosis factor-α (TNF-α) were detected by ELISA. Finally, glucose tolerance and insulin sensitivity were measured by glucose analyzer.

**Results:**

IREB2 was highly expressed in the liver tissue of NAFLD rats. The body weight and liver tissue weight of rats with knockdown of IREB2 were lower than those fed HFD, and the liver tissue was severely damaged, serum ALT/AST activity, glucose, TG, and TC levels were increased. In addition, overexpressing IREB2 increased IL-1β, IL-6, and TNF-α, promoting HFD-induced metabolic disorders, hepatic steatosis, and inflammation. Knocking down IREB2 had the opposite effect. Blocking the TLR4/NF-κB cascade reversed the promoting effect of IREB2 on steatosis and inflammatory response.

**Conclusion:**

NAFLD treatment and prevention could benefit from IREB2, which may be closely related to TLR4/NF-κB signaling in lipid metabolism and glucose tolerance.

**Main Points**

- IREB2 affects liver steatosis and inflammation in nonalcoholic fatty liver disease (NAFLD) rats.
- IREB2 affects glucose tolerance and insulin sensitivity in NAFLD rats.
- IREB2 affects TLR4/NF-κB pathway activation in NAFLD rats.
- Blocking the TLR4/NF-κB pathway reverses the promotion effect of overexpression of REB2 on liver injury and inflammation in NAFLD rats.
- Blocking the TLR4/NF-κB pathway reverses IREB2 overexpression-regulated glucose tolerance and insulin sensitivity in NAFLD rats.

## Introduction

In parallel with obesity, NAFLD has become more prevalent. Nonalcoholic fatty liver disease begins with triacylglycerol accumulation in the liver, which is defined as a fatty infiltration of the liver greater than 5% of liver weight or the presence of more than 5% of hepatocytes containing large fat vacuoles, as well as necrotic inflammatory and cytic changes.^1^ It covers a variety of liver diseases, ranging from steatosis to nonalcoholic steatohepatitis, fibrosis, and hepatocellular carcinoma.^2^,^3^ Current management of NAFLD mainly focuses on interventions through diet and exercise to achieve weight loss and improve underlying metabolic and cardiovascular problems.^4,5^ Although many pharmacological interventions have been tested, including anti-diabetic drugs, anti-dyslipidemia drugs, and anti-obesity drugs^6^ to limit NAFLD development, there is still not a single drug specifically designed to treat NAFLD and control complications.^7-9^ Therefore, a better understanding of lipid accumulation and inflammation in hepatocytes may provide new therapeutic strategies for NAFLD prevention and treatment.

In mammals, iron responsive element binding protein 2 (IREB2) forms part of a group of genes that regulate iron homeostasis. Through iron–sulfur conversion, IREB2 mainly records cytoplasmic iron status.^10^ IREB2 variants are associated with chronic obstructive pulmonary disease (COPD).^11^ There is an increase in IREB2 levels in lung tissue samples from COPD patients, suggesting that IREB2 is a potential new gene associated with COPD susceptibility.^12^ At the same time, lung cancer and COPD may share common genetic and environmental susceptibility factors.^13^ In addition, Liet al^14^ constructed an ischemia–reperfusion injury model of steatohepatitis and an HR model of steatohepatitis cells (SHP-HR). They found that increased expression of IREB2 in steatotic liver insulin resistance (IR) and SHP-HR led to iron death of hepatocytes and further aggravated liver tissue injury. Iron responsive element binding protein 2 has been shown to be a key target leading to liver tissue injury. However, the molecular mechanism details of whether IREB2 is involved in and regulates NAFLD have not yet been discovered.

As a pattern recognition receptor, toll-like receptor 4 (TLR4) is the most characteristic member of the TLR family. Toll-like receptor 4 is closely related to NAFLD, and NAFLD can be prevented by allelic variation of human TLR4.^15^ In addition, the nuclear factor (NF)-κB cascade is known as a major pro-inflammatory switch in NAFLD. Factor transcription in NAFLD is typically regulated by the binding of different homologous or heterodimers, like the p65 and NF-κB consensus sequence formed by NF-κB proteins.^16^ Nuclear factor-κB is believed to be activated as a result of exogenous pathogen-activated TLR4 released by damaged or stressed tissues. This activates the downstream inflammatory cascade and initiates the adaptive immune response, enhancing the production of pro-inflammatory molecules.^17-19^ However, the impact of the TLR4/NF-κB cascade on NAFLD is relatively weak.

Considering that IREB2 was experimentally found to affect the expression of the TLR4/NF-κB pathway, we speculate that IREB2 inhibits NAFLD progression by regulating the TLR4/NF-κB pathway. This study aimed to explore whether IREB2 has a protective effect on NAFLD by regulating the TLR4/NF-κB cascade and to explore the molecular mechanism of IREB2 regulation of NAFLD.

## Materials and Methods

### NAFLD Rat Model

Male Sprague–Dawley rats (180 ± 20 g; 6 weeks old) were purchased from the Laboratory Animal Center at Sanming First Hospital Affiliated to Fujian Medical University Animal Experimental Ethics Committee University of Science and Technology (Fujian, China). All experimental procedures were approved by the Animal Ethics Committee of Sanming First Hospital Affiliated to Fujian Medical University (approval no: 2018SMFH-0310, date: October 15, 2022). The rats were kept under conditions of 22 ± 2°C, 45%-75% relative humidity, and a 12-hour light and dark cycle. After 1 week, the rats were fed either a normal diet (ND, 65% carbohydrates, 23% protein, and 12% fat, based on caloric content) or a high-fat diet (HFD, New Brunswick, NJ, USA; 21% fat, 0.15% cholesterol) for 10 weeks with regular weekly weight measurements.

To produce rats with overexpression of IREB2 in the liver, the IREB2 gene adenovirus (AAV8-IREB2) was injected into rats fed an HFD diet (4 × 10^11^ virus particles/rats). AAV8-green fluorescent protein (AAV8-GFP, Hanheng Biotechnology Co., Shanghai, China) was a negative control. High-fat diet rats were randomly divided into 3 groups: (i) HFD; (ii) HFD + AAV8-GFP; and (iii) HFD + AAV8-IREB2.

Adeno-associated virus serotype 8 (AAV8-IREB2) encodes an RNA construct targeting mouse IREB2. AAV8 vectors encoding codon-optimized mouse IREB2 or green fluorescent protein (GFP) were purified by triple transfection of human embryonic kidney 293 cells under the control of the CMV promoter (AAV8-IREB2 and AAV8-GFP vectors) using a cesium chloride gradient purification protocol.

To generate IREB2-knockdown rats in the liver, IREB2-specific short hairpin RNA adenovirus (AAV8-shRNA-IREB2; WZ Bioscience Inc., Shandong, China) was injected intravenously into rats fed an HFD diet (2.5 × 10^11^ virus particles/rats). AAV8-shRNA-scramble (AAV8-shRNA-sc) was an untargeted control.

To investigate whether the TLR4/NF-κB cascade is involved in and has an effect on NAFLD, the TLR4/NF-κB cascade inhibitor IAXO-102 (3 mg/kg/day, MedChemExpress, New Jersey, NJ, USA) was injected subcutaneously into rats overexpressing IREB2 for 28 days.

After 4 weeks, a pentobarbital sodium injection (60 mg/kg intraperitoneally) was administered to the rats after they had fasted for 12 hours. Blood samples were collected by heart puncture in a tube with or without ethylenediaminetetraacetic acid. By centrifugation (3500 rpm/min) for 10 minutes, plasma and serum were separated and then stored at −80°C. After that, the rats were euthanized by cervical vertebra dislocation. Liver tissue was rinsed with saline, weighed, rapidly frozen (20 mg of liver tissue for oil red O staining), and stored at −80°C.

### Hematoxylin–Eosin Staining

After the liver tissue was fixed in 4% paraformaldehyde (Beyotime, Shanghai, China) for 24 hours, the liver tissue was dehydrated with alcohol and prepared as 5 μm tissue sections, which were permeabilized by paraffin, stained with hematoxylin (G1120, Solarbio, Beijing, China) for 20 minutes, immersed in an acidizing solution for 1 minute, stained with eosin for 15 minutes, dehydrated, and cleared. Finally, the sections were sealed with neutral glue and observed under the optical microscope (CKX31, Olympus).

### Oil Red O Staining

Oil Red O staining kit (C0157S; Beyotime, Shanghai, China) was utilized to check for fatty deposits in the liver. The liver tissue sections (5 μm) were prepared as above and stained with oil red O (positive staining = red fat droplets). The oil red O positive region was quantified in 5 regions, and the percentage was measured using Image-Pro Plus version 4.5 (Media Cybernetics, Maryland, MD, USA).

### Measurement of Serum Insulin

Serum insulin was determined by an enzyme-linked immunosorbent assay (ELISA) kit (Kusa BioBiotechnology Co., LTD., Wuhan, China).

### Intraperitoneal glucose tolerance test (IPGTT) and intraperitoneal insulin tolerance test (IPITT)

For IPGTT, rats were fasted for 14 hours, and blood samples were collected from the tail tip. Rats were given intraperitoneal injections of 25% glucose solution (1 mg/g, Beyotime, Shanghai, China), and after 30, 60, 90, and 120 minutes, fasting blood glucose (FBG) was measured using a 1-click glucose meter (Lifescan, California, CA, USA). For IPITT, rats were fasted for 4 hours, and blood samples were taken as described above. Insulin was injected intraperitoneally (0.75 mU/g; Novo Nordisk, Beijing, China), and blood glucose was measured at 15, 30, 45, and 60 minutes. Measurements were performed every 2 weeks.

### Biochemical Analysis

Serum total cholesterol (TC), triglycerides (TG), alanine aminotransferase (ALT), and aspartate aminotransferase (AST) were measured using a biochemical automatic analyzer (Fuji Medical System, Tokyo, Japan).

### Inflammatory Indicators

Serum concentrations of interleukin-1-beta (IL-1β), Interleukin-6 (IL-6), and tumor necrosis factor-α (TNF-α) were measured using ELISA kits (Tsz Biosciences, MA, USA).

### Quantitative Reverse Transcription Polymerase Chain Reaction

Total RNA was isolated from liver tissue using TRIzol reagent (Invitrogen, California, USA). Gene assay was conducted with PrimeScript RT Reagent Kit (Takara, Shanghai, China) and ABI 7500 real-time fluorescent quantitative PCR system (Applied Biosystems, Foster City, CA, USA) and SYBR Premix Ex Taq Kit (Takara, Shanghai, China). The Applied Biosystems 7500 Real-Time PCR System was subjected to 40 cycles of 95°C for 30 minutes, 60°C for 60 seconds, and 72°C for 30 seconds. Specific primers designed are shown in Table 1. IREB2 expression was calculated by the 2^−ΔΔCT^ method and standardized by GAPDH.

### Western Blot Assay

To isolate total proteins, the liver tissue underwent 2 washes with frozen phosphate buffered saline and was then lysed using radioimmunoprecipitation assay lysis buffer with protease and phosphatase inhibitors (Roche, Basel, Switzerland) for a duration of 30 minutes. After measuring the protein concentration with the BCA Protein Assay Kit (Beyotime, Shanghai, China), proteins were separated on a 10% SDS-PAGE gel, transferred to a polyvinylidene fluoride membrane (Millipore, USA), and then blocked using 5% skim milk for 2 hours. At 4°C, β-actin (#MAB1501; Millipore), IREB2 (PA1-16544, ThermoFisher, Shanghai, China), TLR4 (ab217274, Abcam, Shanghai, China), p-p65 (ab16502, Abcam, Shanghai, China), fatty acid synthase (Fas; ab82419, Abcam, Shanghai, China), and PPARα (MA1-822, ThermoFisher, Shanghai, China) were incubated overnight. Then, anti-rat IgG coupled with horseradish peroxidase was reacted at room temperature for 6 hours. The images were developed using the Chemo Dox XRS system (Bio-Rad, CA, USA) and analyzed by ImageJ version 6.0.

### Immunohistochemistry

Liver tissue sections (5 mm) were prepared as described above, dewaxed, and treated with 3% hydrogen peroxide for 15 minutes. Then, the sections were sealed with 5% normal serum for 30 minutes and detected with primary antibodies (IREB2 and TLR4) at 4°C and a secondary antibody. Images were obtained through an optical microscope (CKX31, Olympus) and analyzed by ImageJ 6.0.

### Data Analysis

SPSS version 20 (IBM SPSS Corp.; Armonk, NY, USA) statistical software was utilized to analyze the experimental data. The measurement data were expressed as mean ± SD and analyzed by *t*-test or one-way analysis of variance. *P < .05 signified a statistical difference.

## Results

### IREB2 Affects Liver Steatosis and Inflammation in NAFLD Rats

**Figure 1.**
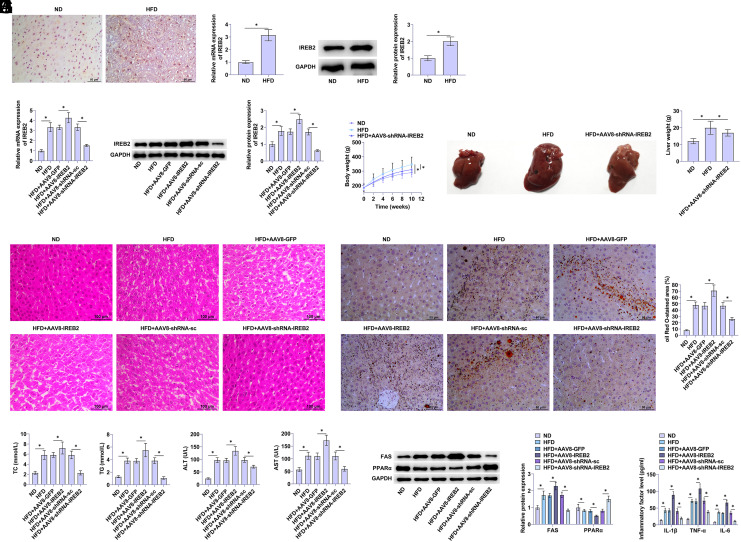
IREB2 affects liver steatosis and inflammation in NAFLD rats. A: IHC staining of IREB2 in NAFLD rat liver tissue. B: PCR and western blot detected IREB2 in rats. C: PCR and western blot detected IREB2 after adenovirus injection. D: Changes in body weight of rats. E: Changes in liver tissue weight. F: Hematoxylin–eosin staining of rat liver tissue. G: Oil red O staining of fat droplets. H: TC, TG, ALT, and AST in rat serum. I: Western blot analysis detected FAS and PPARα. J: ELISA analyzed IL-1β, IL-6, and TNF-α concentrations. Data are expressed as mean ± SD (n = 3). *P < .01.

To determine IREB2’s role in liver lipid metabolism, IREB2 expression was examined in the liver tissue of rats fed an HFD. IREB2 expression was forced in NAFLD liver tissues by IHC assay (Figure 1A). In addition, HFD significantly elevated IREB2 expression in the liver tissue of rats (Figure 1B). After HFD rats were infected with AAV8-IREB2 and AAV8-IREB2-shRNA adenoviruses, PCR and western blot analysis showed that IREB2 in liver tissues was up-regulated or knocked down, respectively (Figure 1C), indicating successful adenovirus interference. After healthy rats were fed an HFD diet, body and liver weight increased significantly, while the amount of fat around the liver tissue increased. The body and liver weights of rats fed an ND remained at normal levels. Knockout of IREB2 inhibited the increase of body weight and liver tissue weight in NAFLD rats (Figure 1D and E).

Liver steatosis and inflammation were evaluated using hematoxylin–eosin staining. When HFD was given, liver tissue was damaged due to severe steatohepatitis, a large amount of fat accumulated around the liver tissue due to inflammation, hepatic sinuses were significantly congested and dilated, and non-structural coagulation necrosis was observed (Figure 1F). The assessment of liver fat droplets was conducted using oil red O staining. The HFD group exhibited notable lipid accumulation and widespread red particle distribution, with a greater proportion of the oil red O staining area, signifying the successful formation of a severely steatotic liver. Overexpressing IREB2 aggravated steatosis in the form of inflammatory infiltration, increased the size of fat droplets in the liver, and promoted increased lipid accumulation. In contrast, knocking down IREB2 reduced lipid accumulation (Figure 1G). High-fat diet-induced TC, TG, ALT, and AST. When IREB2 was overexpressed, TC and TG levels were promoted, while ALT and AST activities were enhanced (Figure 1H). Subsequently, to investigate fatty acid metabolism, molecules involved in fat production and breakdown were analyzed. In rats fed HFD, overexpressing IREB2 elevated FAS and reduced PPARα proteins. Knocking down IREB2 showed the opposite effect (Figure 1I).

Given that obesity is a long-term inflammation potentially harming the liver, the presence of IL-1β, IL-6, and TNF-α in liver inflammation was identified using the ELISA kit. High-fat diet led to increased serum IL-1β, IL-6, and TNF-α, and down-regulating IREB2 reduced serum inflammation after HFD induction, contrary to the effect of overexpressing IREB2 (Figure 1J).

### IREB2 Affects Glucose Tolerance and Insulin Sensitivity in NAFLD Rats

**Figure 2.**
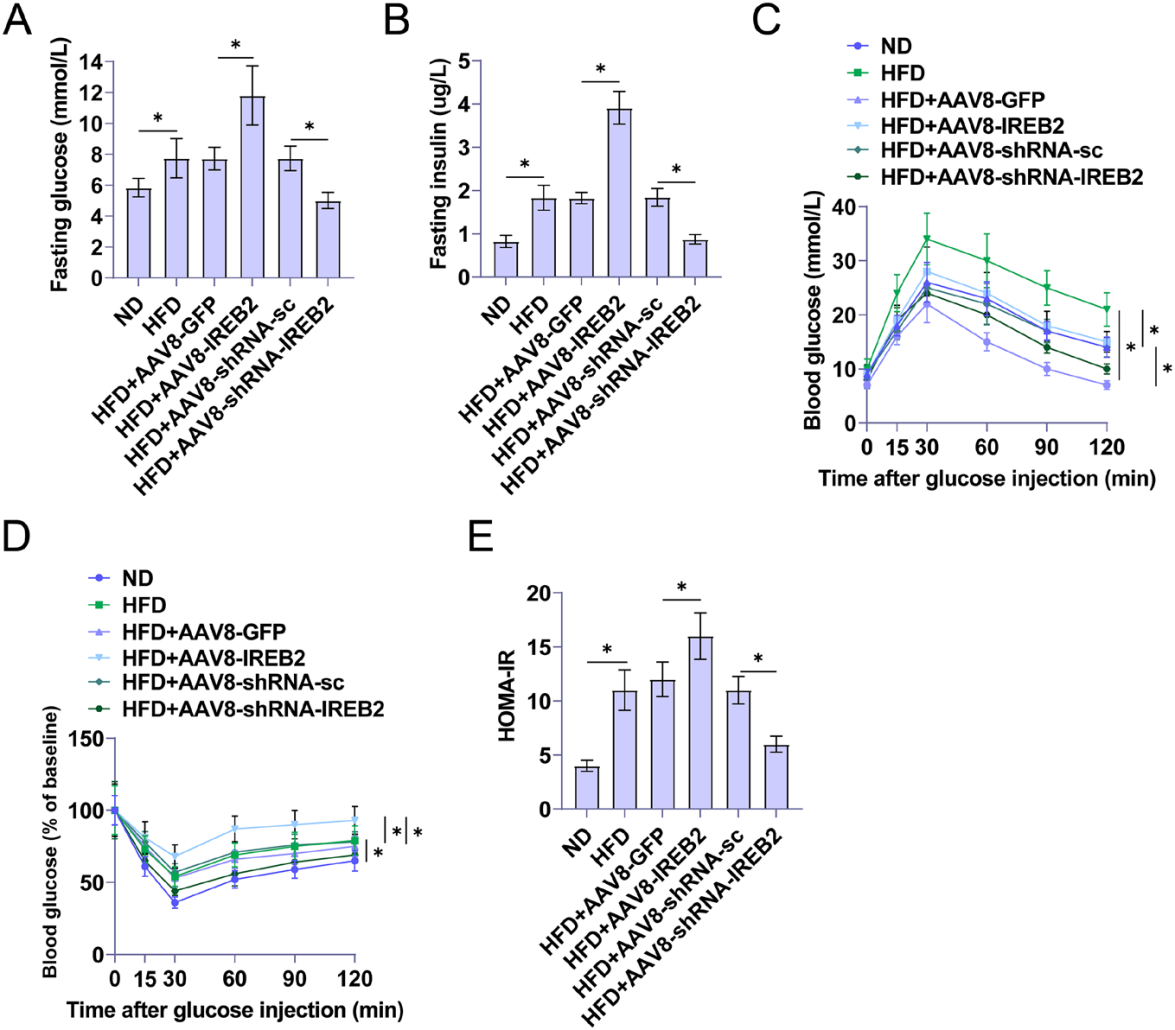
IREB2 affects glucose tolerance and insulin sensitivity in NAFLD rats. A: FBG level of rats. B: Plasma insulin levels. C: Glucose tolerance test. D: Insulin tolerance test. E: HOMA-IR calculated based on FBG and insulin levels. Data are expressed as mean ± SD (n = 3). *P < .05.

Glucose homeostasis and insulin sensitivity in rats were assessed using IPGTT and IPITT to understand whether IREB2 alters IR. Results showed increased FBG and insulin levels in HFD-induced rats. Upregulating IREB2 further elevated FBG and insulin levels, with decreased FBG and insulin levels shown after IREB2 knockdown (Figure 2A and B). High-fat diet-fed rats after IREB2 overexpression showed an enhanced response to glucose tolerance (Figure 2C). Consistently, a rapid glucose response to insulin load was not evident in HFD-fed rats after overexpressing IREB2 (Figure 2D), inhibiting insulin sensitivity while increasing the Homeostatic Model Assessment for Insulin Resistance (HOMA-IR) score (Figure 2E). In contrast, knockdown of IREB2 inhibited glucose tolerance and HOMA-IR.

### IREB2 Affects TLR4/NF-κB Cascade Activation in NAFLD Rats

**Figure 3.**
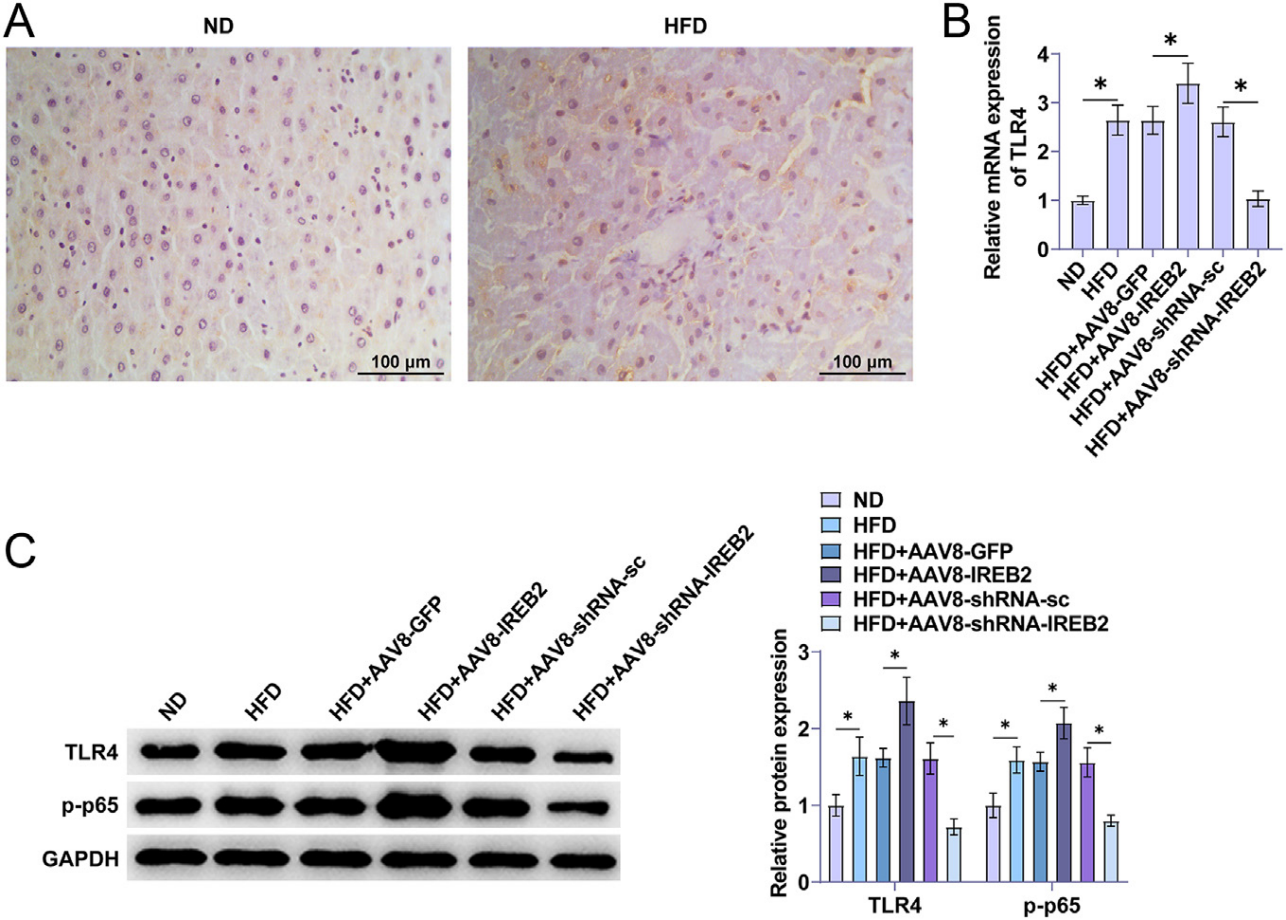
IREB2 affects TLR4/NF-κB cascade activation in NAFLD rats. A: IHC staining of TLR4 in liver tissue of NAFLD rats. B: RT-qPCR detection of the expression level of TLR4. C: Western blot to determine the protein expression levels of TLR4 and p-p65. Data are expressed as mean ± SD (n = 3). *P < .01.

To explore whether IREB2 regulates the TLR4/NF-κB cascade in NAFLD, IHC assays showed high expression of TLR4 in NAFLD liver tissue (Figure 3A). In addition, the quantitative reverse transcription polymerase chain reaction (RT-qPCR) assay showed that overexpression of IREB2 promoted TLR4 expression in NAFLD, and down-regulation of IREB2 showed the opposite effect (Figure 3B). Western blot analysis showed that TLR4 and p-p65 proteins were elevated in NAFLD rats, whereas overexpression of IREB2 promoted the expression of TLR4 and p-p65, and knockdown of IREB2 could inhibit the levels of TLR4 and p-p65 proteins (Figure 3C).

### Blocking the TLR4/NF-κB Cascade Reverses the Promotion Effect of Overexpression of REB2 on Liver Injury and Inflammation in NAFLD Rats

**Figure 4.**
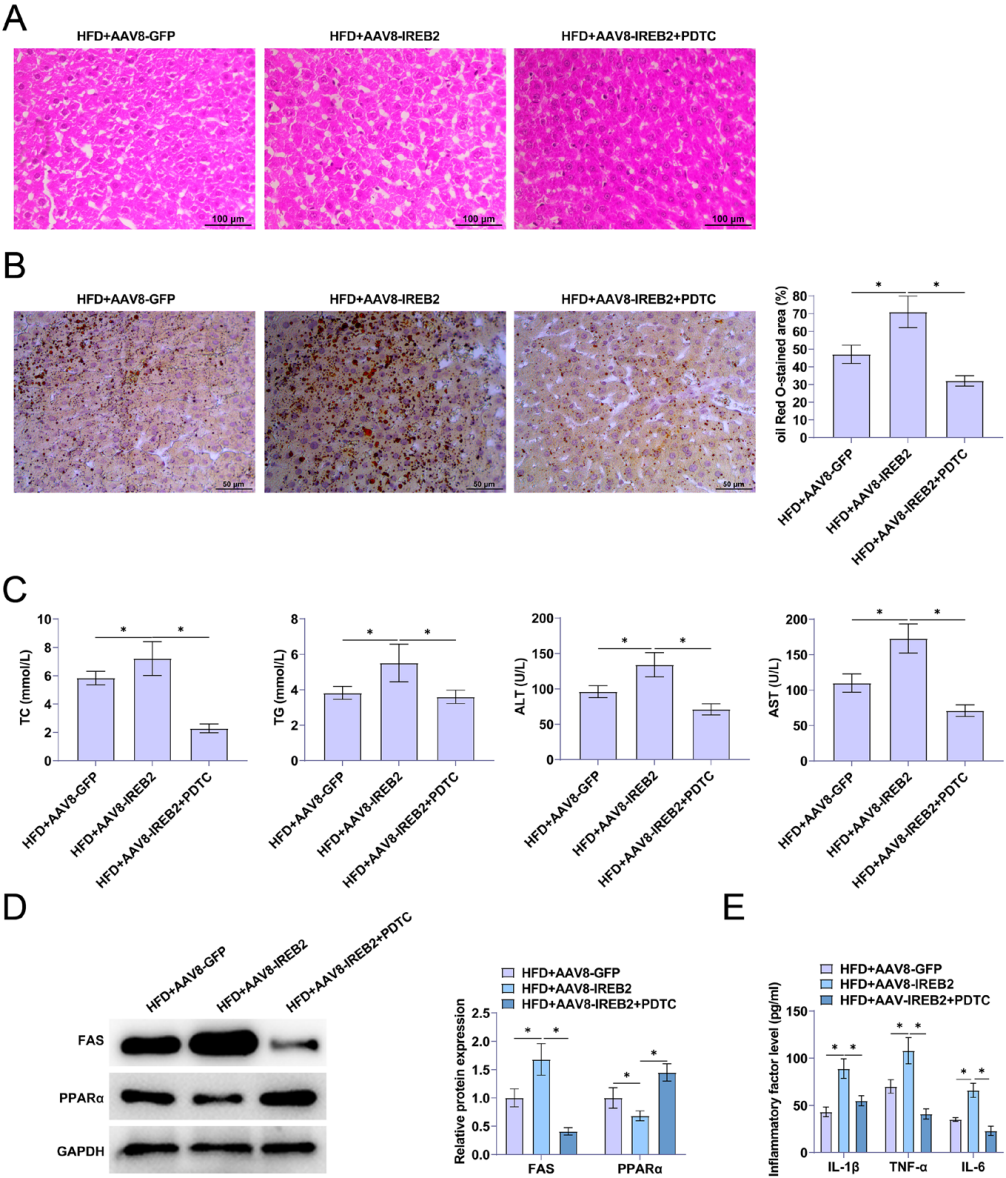
Blocking the TLR4/NF-κB cascade reverses the promotion of overexpression of IREB2 on liver injury and inflammation in NAFLD rats. A: Hematoxylin–eosin staining of rat liver tissue. B: Oil red O staining of rat liver tissue. C: TC, TG, ALT, and AST in rat serum. D: Western blot analysis detected FAS and PPARα. E: ELISA detected IL-1β, IL-6, and TNF-α concentrations.

Pyrrolidine dithiocarbamate (PDTC), an inhibitor of the TLR4/NF-κB cascade, was administered to NAFLD rats injected with the AAV8-IREB2 adenovirus to block the TLR4/NF-κB cascade. Pyrrolidine dithiocarbamate treatment reduced the degree of liver tissue damage caused by overexpression of IREB2 and effectively improved microvesicular steatosis (Figure 4A). Blocking the TLR4/NF-κB cascade following IREB2 overexpression decreased the percentage of oil red O stained areas (Figure 4B). Pyrrolidine dithiocarbamate mitigated the promoting effect of IREB2 on TC, TG, ALT, and AST and inhibited the disorder of lipid metabolism (Figure 4C). Pyrrolidine dithiocarbamate rescued the influence of IREB2 overexpression on FAS and PPARα proteins (Figure 4D). Moreover, PDTC decreased IL-1β, IL-6, and TNF-α (Figure 4E) and reversed the promoting effect of upregulated IREB2 on inflammation.

### Blocking the TLR4/NF-κB Cascade Reverses IREB2 Overexpression-regulated Glucose Tolerance and Insulin Sensitivity in NAFLD Rats

**Figure 5.**
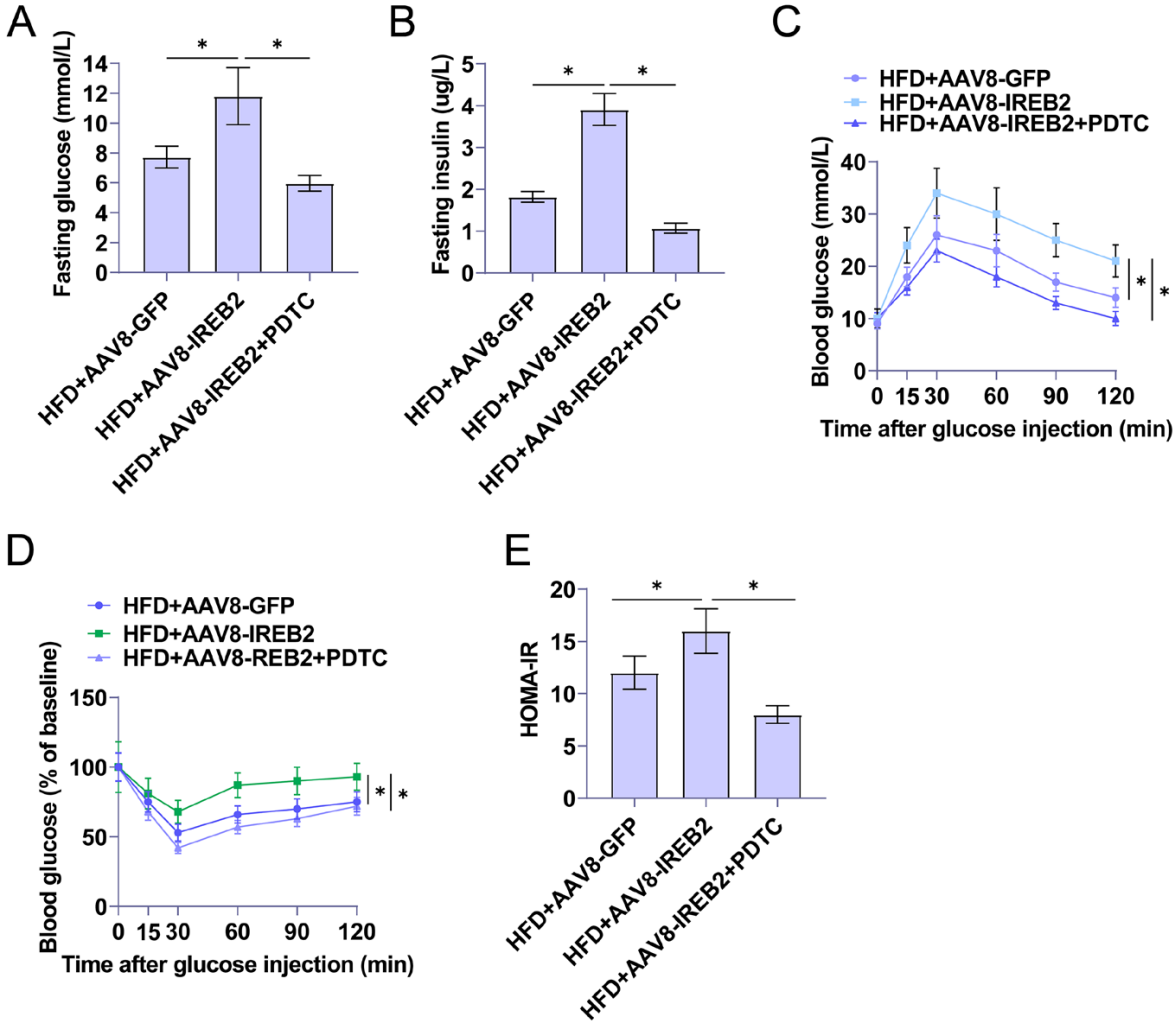
Blocking the TLR4/NF-κB cascade reverses IREB2 overexpression-regulated glucose tolerance and insulin sensitivity in NAFLD rats. A: FBG level of rats. B: Plasma insulin levels. C: Glucose tolerance test. D: Insulin tolerance test. E: HOMA-IR calculated based on FBG and insulin levels.

Pyrrolidine dithiocarbamate significantly reduced FBG and insulin levels in rats with overexpressed IREB2 (Figure 5A and B). In addition, blocking the TLR4 /NF-κB cascade significantly reduced the glucose tolerance response in rats with overexpressed IREB2 (Figure 5C). The rapid response to insulin load was improved after PDTC treatment (Figure 5D) and the HOMA-IR score was decreased (Figure 5E).

## Discussion

Globally, NAFLD, defined as the accumulation of pathological TG in hepatocytes, is prevalent among chronic liver diseases^20^ and has many complex biochemical, metabolic, and clinical manifestations. The earliest stage of NAFLD is steatosis, which is characterized by increased production of new fat and the accumulation of large amounts of TG in the liver, which, if out of control, can develop into hepatitis or cirrhosis.^21^ At the same time, high levels of plasma TG and saturated fatty acids promote the secretion of inflammatory and pro-inflammatory cytokines associated with metabolic syndrome, triggering inflammatory responses.^22^ In this study, inhibition of the TLR4/NF-κB signaling pathway activation reversed the promotional effect of overexpression of IREB2 on hepatic tissue injury and inhibited hepatic steatosis deformation and inflammatory response in NAFLD rats.

Fe^2+^ is a cause of steatosis liver and hepatic IR, and it is possible to slow down NAFLD and hepatic IR by inhibiting ferroptosis,^23,24^ and increased iron load and Fe^2+^ levels are usually found during NAFLD liver IR.^25,26^ The ferroptosis marker gene IREB2 increases its mRNA expression as ferroptosis occurs. Therefore, we hypothesized that IREB2 plays an important role in NAFLD liver tissue degeneration and IR. In the present study, we used male rats to carry out the experiments. Ganz et al^27^ found sex differences in the development of fatty liver degeneration and inflammatory factor activation in NAFLD, and in female mice, only an increase in triglycerides was observed without steatohepatitis. Kamada et al^28^ showed that estrogen plays a role in the protection against liver injury after a HFD, with the incidence of NAFLD reduced. Moreover, according to a previous study, extensive use of MCD resulted in severe NAFLD in a short period of time, but animal models showed weight loss and reduced IR.^29^ For better induction of NAFLD in anin vivo model, HFD feeding was used, and liver tissue showed fibrosis after 6 weeks. IREB2 was highly expressed in NAFLD, and overexpressing IREB2 significantly promoted the malignant development trend of NAFLD rats, leading to serious liver tissue damage and lipid deposition. In addition, overexpressing IREB2 increased the deposition of TC and TG, and the activities of ALT and AST, thus promoting a disorder of lipid metabolism. PPARα is considered to be a major factor regulating fatty acid β-oxidation, which can promote the intake of fat and mitochondrial fatty acid oxidation.^30,31^ PPARα deficiency aggravates hepatic steatosis in NAFLD by weakening fatty acid oxidation.^32^ FAS is responsible for regulating TG synthesis in the liver. FAS protein increased and PPARα protein decreased in NAFLD rats. A systemic chronic low inflammation is at the core of obesity.^33^ To investigate whether IREB2 is involved in the inflammatory response in NAFLD, we also looked at changes in inflammatory cytokines. Upregulated IREB2 increased IL-1β, IL-6, and TNF-α in rats, while knocking down IREB2 appeared to have the opposite effect. Overexpressing IREB2 promoted glucose tolerance and inhibited insulin sensitivity.

In NAFLD, gram-negative bacteria and ROS induce TLR4 activation. The TLR4/NF-κB cascade in hepatocytes enhances the release of inflammatory cytokines and promotes chronic inflammation.^34^ In obese patients, activation of NF-κB promotes the expression of inflammatory cytokines, and the massive release of inflammatory factors such as IL-6 and TNF-α further enhances NF-κB activity. These gene products regulated by NF-κB are involved in chronic inflammation and liver damage in liver tissue.^35^ The same trend was expressed in our study. Importantly, TNF-α has a strong inhibitory effect on lipoprotein lipase, which reduces catabolism of peripheral adipose tissue, promotes TG synthesis in hepatocytes, and induces lipid accumulation in the liver.^36^ In NAFLD rat models, PDTC reversed the promoting effect of IREB2 on liver injury and inflammation in rats, inhibited fat droplet formation in the liver, lowered TC, TG, ALT, and AST, and suppressed fat accumulation. Tumor necrosis factor-α can impede insulin signaling by inducing the expression of signal transduction inhibitor 3, leading to IR.^37^ Thus, PDTC decreased IL-1β, IL-6, and TNF-α, weakened glucose tolerance, and improved insulin sensitivity. Thus, the TLR4/NF-κB signaling pathway plays an important role in NAFLD progression and, consistent with previous findings, regulates the expression of IL-6 and TNF-α, key factors involved in NAFLD development.

Regarding the limitations of the present study, we did not perform the analysis of clinical data, and the protein levels of IREB2 should be verified in liver tissues of NAFLD patients and healthy normal control subjects. The effect of IREB2 on Kupffer cells, the most important macrophages in the liver, is not known. In addition, many factors regulate iron death, and this study focused on only a few typical iron death markers to determine the effects of IREB2 and TLR4/NF-κB on iron death, but it is possible that changes in other factors that we did not study occurred.

In summary, IREB2 leads to enhanced lipid accumulation, liver inflammation, and liver tissue damage in an HFD-induced NAFLD rat model. In addition, this study provided a mechanism by which IREB2 regulates lipid metabolism and glucose tolerance in NAFLD rats through the TLR4/NF-κB cascade. A novel target for NAFLD prevention and treatment is considered to be IREB2, which regulates the TLR4/NF-κB cascade in HFD-induced NAFLD.

## Figures and Tables

**Table 1.** Primer Sequence

Gene	Forward Primer (5’ →3’)	Reverse Primer (5’ →3’)
IREB2	CATCAGGACAGACGCTCGATG	CAGCCAAAACAGCCTTTACACC
TLR4	AGAGAATCTGGTGGGTGGGTGGAGAC	AAAGGCTTGGGCTTGAATGGAGTC
GAPDH	GTCCATGCCATCACTGCCACTC	CGCCTGCTTCACCTTCTTG

IREB2, iron responsive element binding protein 2; TLR4, toll-like receptor 4; GAPDH, glyceraldehyde 3-phosphate dehydrogenase.
